# A validated HPLC-MS/MS method for the simultaneous determination of ecdysteroid hormones in subminimal amounts of biological material

**DOI:** 10.1016/j.jlr.2024.100640

**Published:** 2024-09-05

**Authors:** Lucie Marešová, Martin Moos, Stanislav Opekar, Michalina Kazek, Clemens Eichler, Petr Šimek

**Affiliations:** 1 Laboratory of Analytical Biochemistry and Metabolomics, Biology Centre, Czech Academy of Sciences, České Budějovice, Czech Republic; 2 Department of Chemistry of Natural Compounds, Faculty of Food and Biochemical Technology, University of Chemistry and Technology Prague, Prague, Czech Republic; 3 Faculty of Health and Social Sciences, Institute of Laboratory Diagnostics and Public Health, University of South Bohemia, České Budějovice, Czech Republic

**Keywords:** ecdysteroid hormones, submilligram sample amount, ultratrace HPLC-MS analysis, quantification, arthropods, human body fluid, dietary supplementation

## Abstract

Ecdysteroids represent a large class of polyhydroxylated steroids which, due to their anabolic properties, are marketed as dietary supplements. Some ecdysteroids also act as important hormones in arthropods, where they regulate molting, development, and reproduction and many of these insects are miniature organisms that contain submicroliter levels of circulating biofluids. Analysis of ecdysteroids is further complicated by their very low abundance, large fluctuations during development, and difficult access to a pooled sample, which is important for quantitative measurements. In this work, we propose a new method that overcomes the described difficulties and allows validated quantification of four ecdysteroids in minimal amounts of biological material. After methanolic extraction, detectability of the ecdysteroids is increased 16- to 20-fold by conversion to their 14,15-anhydrooximes. These are further purified by pipette tip solid-phase extraction on a three-layer sorbent and subjected to HPLC-MS/MS analysis. Full validation was achieved using hemolymph from larvae of the firebug *Pyrrhocoris apterus* as a blank matrix and by the determination of ecdysteroids in a single *Drosophila* larva. The lower limit of quantifications for the four target ecdysteroids (20-hydroxyecdysone, ecdysone, makisterone A, and 2-deoxyecdysone) were 0.01; 0.1; 0.05; and 0.025 pg·ml^-1^ (20; 200; 100; 50 fmol ml^-1^), respectively, with very good accuracy, precision (expressed as relative standard deviation <15%) and recoveries (96%–119.9%). The application potential of the new method was demonstrated by quantification of ecdysteroids in various biological materials including human serum.

## Graphical abstract

**Figure:**
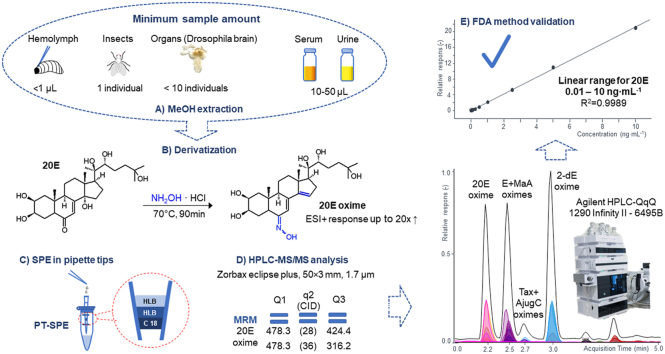

In the animal kingdom, many steroids are important hormones that circulate throughout the body as signaling molecules and regulate the physiology and behavior of individual organisms. Ecdysteroids belong to the class of polyhydroxylated steroids that have an interesting variety of modalities (1, 2, 3, 4). In humans, but also in domestic mammals such as equids, ecdysteroids exhibit natural anabolic properties that increase physical performance and facilitate recovery, which is why they are marketed as dietary supplements (5, 6). Some plants, such as *Leuzea carthamoides* (also known as maral root) or the Chinese plant *Cyanotis arachnoidea* (7, 8, 9), have a relatively high content of ecdysteroids (phytoecdysteroids), especially 20-hydroxyecdysone (20E). Extracts from plants rich in ecdysteroids are produced as dietary supplements and are reported to have various health benefits, including stimulation of the immune system, positive effects on carbohydrate and fatty acid metabolism, and stimulation of protein synthesis. In the taxonomic superphylum group Ecdysozoa, ecdysteroids act as hormones that regulate several important biochemical processes, particularly molting, development, and reproduction. Insects synthesize ecdysteroids from dietary cholesterol, probably in the prothoracic gland (10, 11). 20E is the most abundant and also the most biologically active ecdysteroid (12), followed by makisterone A (MaA), ecdysone (E), 2-deoxyecdysone (2dE) and some minor ecdysteroids such as 20-deoxymakisterone A, 24-epi-makisterone A, and 24,28-dehydromakisterone A (11, 13).

Ecdysteroids circulate in the insect body at the femtomole level and are difficult to measure in complex biological material. Compared to vertebrates, many arthropods (especially insects) are tiny organisms that contain very small, often submicroliter volumes of circulating biofluids in their bodies. In addition, analysis is complicated by large fluctuations in hormone levels (as shown in peak profiles) that can range from 0 to about 0.2 pmol per individual (14), reflecting an even smaller amount of ecdysteroids in the circulating biofluid. The individual development of each organism does not allow the use of a pooled sample needed for matrix calibration and subsequent full method validation according to established guidelines (15, 16). After reviewing the current literature, we find no robust analytical method capable of accurately quantifying ultratrace levels of these hormones in a minimal amount of available biological material such as arthropod biofluids.

The first methods for quantification of ecdysteroids in the ultratrace range were radioimmunoassays and bioassays, often coupled with chromatographic separation such as TLC or HPLC, which improved the sensitivity and selectivity of the method (13, 17, 18). However, these approaches lack specificity as they only provide information on total content, as it is not possible to distinguish between individual ecdysteroids, and cross-reactivity with nonecdysteroids may also occur (19, 20). More specific GC-MS requires a substantial increase in the volatility of the steroid analytes by silylation of the numerous sterically hindered hydroxyl groups present, resulting in water-sensitive derivatives and frequently occurring by-products (21, 22), making their selective and robust quantification difficult. Current analytical approaches are based on workflows involving extraction and enrichment of ecdysteroids by solid-phase extraction (SPE) and their subsequent LC-MS analysis. Triple quadrupole mass analyzers (QqQ) operated in multiple reaction monitoring (MRM) MS/MS mode are generally used (5, 6, 7, 11, 13, 21, 23, 24, 25, 26, 27). Since the ecdysteroids are not efficiently ionizable by electrospray, their carbonyl group was also derivatized by Girard reagents (28) or hydroxylamines (29, 30) to increase the MS response.

In this study, we aimed to develop a novel, robust, fully validated LC-MS/MS method capable of measuring ecdysteroid titers in very small amounts of biological material, even in submicroliter volumes of a biofluid that can often be obtained from the body of a single animal. The list of all ecdysteroids investigated and their derivatives, with structural information, common name, and Chemical Abstracts Service number is shown in Fig. 1.

**Fig. 1:**
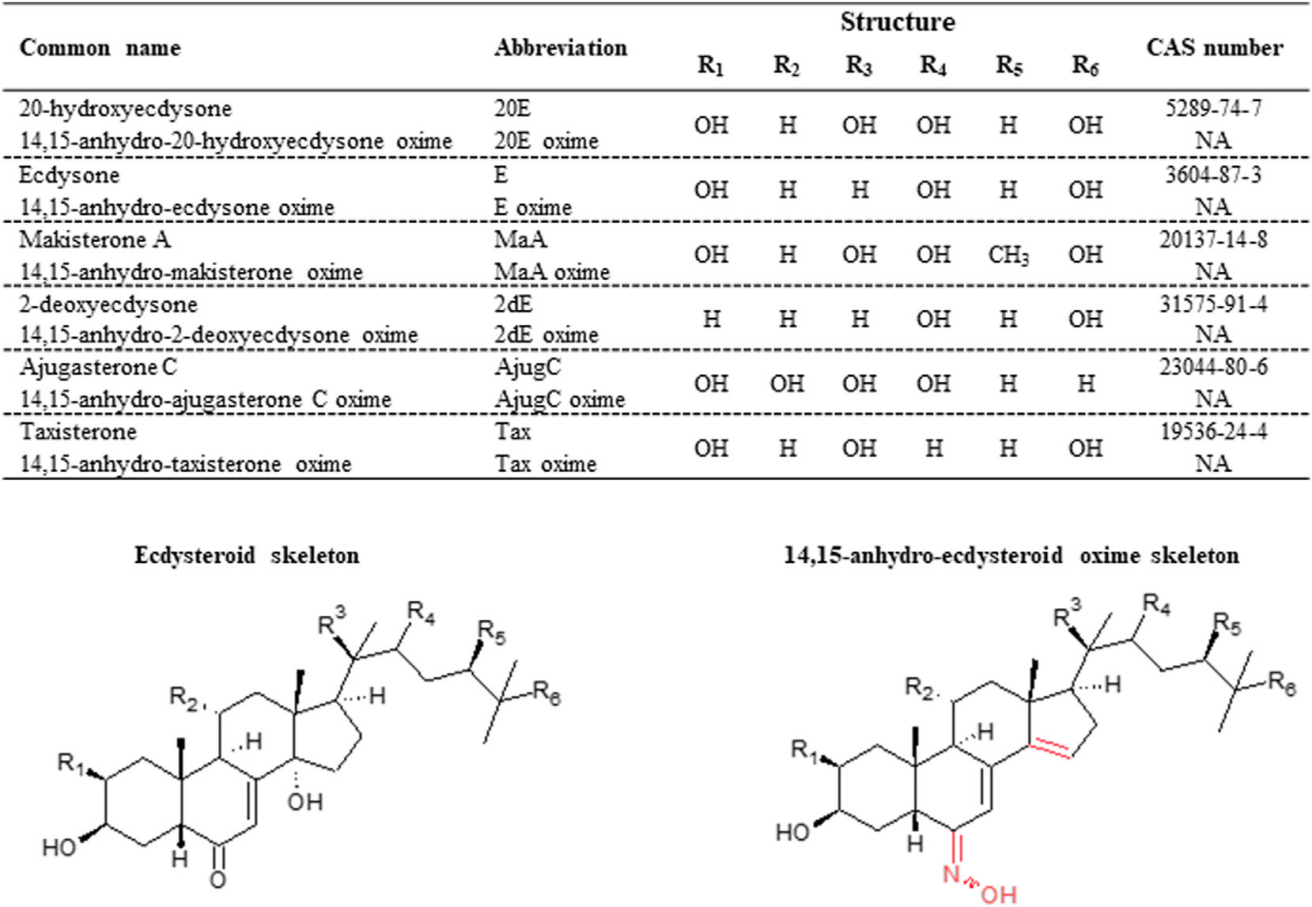
Summary of the investigated ecdysteroids and their oxime derivatives, which are particularly important for the life cycle of insects. The structures represent the ecdysteroid skeleton and the skeleton of ecdysteroids derivatized by hydroxylamine, which forms the respective oxime. The table contains the full names, abbreviations used, structural details and Chemical Abstracts Service number where available. For 20-hydroxyecdysone the term “ecdysterone” is often used, especially in older literature.

The workflow combining the steps of methanol (MeOH) extraction, hydroxylamine derivatization, pipette tip SPE (PT-SPE), with positive ESI^+^ MRM LC-MS/MS analysis, was validated for ultratrace quantification of four major ecdysteroids in 1 μl of hemolymph biofluid from a single *Drosophila* larval body according to the Food and Drug Administration (FDA) guidelines for bioanalytical methods (15). The performance of the newly presented method was further evaluated for ecdysteroid analysis in various biological materials, including human serum and peripheral blood.

## Materials and Methods

### Chemicals, reagents, and materials

20E (97% purity, Toronto Research Chemicals, Toronto, Canada), ecdysone, and MaA standards (both ≥95% purity) were purchased from Santa Cruz Biotechnology (Dallas, TX). Ajugasterone C (>98% purity) was obtained from Wuhan ChemFaces Biochemical (Wuhan, PRC). 2-deoxyecdysone was a generous gift from Dr Juraj Harmata (Institute of Organic Chemistry and Biochemistry, Prague,). Each ecdysteroid standard was dissolved in MeOH at a stock concentration of 1 mg ml^−1^. Reserpine (98% purity), the injection standard, was purchased from Sigma-Aldrich (St. Louis, MO). Hydroxylamine hydrochloride (NH_2_OH·HCl), the derivatizing agent, was purchased from Merck (ReagentPlus, 99% purity). MeOH (HPLC-MS grade) was obtained from VWR (Radnor, PA). Deionized water was prepared using the Divert-Q ®UV water purification system (Merc, Darmstadt, Germany). Grounded “maral” root extract from *Leuzea carthamoides* was obtained from a local pharmacy supplier.

### Synthesis of 20E-oxime

For the synthesis of 20E-oxime from 20E and hydroxylamine hydrochloride, a procedure from the literature was used (30). In brief, 20E (125 μmol; 70 mg; 1 eq.) was dissolved in freshly distilled pyridine (2.0 ml). Hydroxylamine hydrochloride (0.875 mmol; 60 mg; 6 equiv.) was added and the mixture was heated and stirred in a closed vessel at 70°C for 72 h. After cooling to 0°C the mixture was concentrated under reduced pressure. Water (5 ml) was then added and extracted three times with ethyl acetate (8 ml). The organic phase was collected and dried with Na_2_SO_4_. After filtration, the ethyl acetate was evaporated. The residue obtained was subjected to silica gel column chromatography (chloroform-MeOH 7:1), which yielded 45 mg (76%) of colorless solid: the retention factor (R_f_) was 0.30 (vanillin stain detection).

The NMR spectrum of the synthesized oxime (prepared for recovery calculations of the sample preparation procedure) was recorded on a Bruker Avance III 400. Spectra for 1 H at 400.13 MHz were recorded in deuterated pyridine at the ambient temperature. ACD/NMR processor Academic Edition 12.0 (ADC/Labs, Toronto, Canada) was used to process the NMR data. The ^1^H and ^13^C NMR spectra corresponded to the published values of the *E*- and *Z*-mixture of 14,15-anhydro-20-hydroxyecdysone-6-oxime (30). The HRMS (ESI^+^) spectrum, *m/z*: calculated for C_27_H_43_NO_6_, [M + H]^+^ = 478.3163, found 478.3161.

### Insect growing and collection of biological samples

Larvae of *Drosophila melanogaster* (Diptera) with genetic background *w*^*1118*^ were used for the study. Flies were reared on standard cornmeal medium (8% cornmeal, 5% glucose, 4% yeast, and 1% agar) at 25°C. Before hemolymph extraction, *Drosophila* larvae were first washed with distilled water and then with PBS (from Merc, Darmstadt, Germany) to sterilize their surfaces and reduce contamination. Larval hemolymph was collected by carefully spreading the larvae onto glass microscope slides covered with parafilm. Fresh hemolymph was immediately transferred to sterile 1.5 ml Eppendorf polypropylene tubes filled with 50 μl of cold MeOH. To obtain hemolymph from prepupa, pupa, or adult flies, animals were placed in 0.5 ml tubes, punctured with forceps, and centrifuged at 9500 *g* for 3 min at 4°C. Then, the hemolymph was transferred to a new 1.5 ml Eppendorf tube containing cold MeOH. All experimental steps were performed on ice to prevent melanization of the hemolymph.

The day-old adults of the fire bug *Pyrrhocoris apterus* (Heteroptera) used in the present study were mass-cultured in 0.5-l jars (approximately 40 specimens per jar) and reared at a constant temperature of 26 ± 1°C under long-day conditions (18:6 h light:dark). They were supplied ad libitum with lime seeds and water. Hemolymph was collected from the cut antenna. The exuding hemolymph droplets were collected on a piece of parafilm and then pipetted into a 1.5 ml Eppendorf vial containing cold MeOH for further use. Other arthropod species examined, that is, adults of *Ixodes ricinus* and larvae of *Pityiogenes chalcographus* as well as pupae and larvae of *Culex quienquefasciatus*, originated from in-house rearings.

### Human serum, urine, and peripheral capillary blood

The second-void morning urine, plasma, and peripheral capillary blood from fingerstick prick samples were obtained upon written informed consent from seven healthy adult laboratory participants (three men and four women aged 30–67 years) who regularly attended health check-ups in the České Budějovice hospital. The study abided by the Declaration of Helsinki principle and was approved by the Local Research Ethics Committee (České Budějovice hospital, ethic number LEC 103/20). The participants stated that they had no obvious disease or metabolic disorder. Subjects consumed one tablespoon (approximately 1.5 g) of powdered maral root (*Leuzea carthamoides*), a dietary supplement rich in 20E, in the morning and the afternoon for 14 days. Three subjects did not consume the maral root and served as controls.

### Preparation of the PTs-SPE

The PTs-PSE were filled manually according to a procedure originally described by Rappsilber *et al*. (31) for proteomic applications. A small piece of the excised SPE disk (Affinisep, Petit Couronne, France) containing a sorbent enmeshed into an inert polytetrafluoroethylene carrier was used. The tools required for the preparation process are shown in supplemental Figure S1.

The extraction disk was placed on a clean Petri dish and a small part of the disk (about 1 mm in diameter) was cut out with a specially modified needle from a Hamilton syringe, which remained in the needle (cutter). The cutter with the sorbent inside was then inserted into a standard 200 μl PT (Eppendorf Hamburg, Germany), and the sorbent was squeezed out with the plunger (modified piston from another Hamilton syringe) and dispensed into the tip. Again, using the plunger, the sorbent was carefully pushed to the bottom of the tip. To create another layer of sorbent in the SPE tip, the procedure was repeated with another piece of the disk containing the same or a different sorbent.

The finished SPE tip was placed through the hole in the lid into a 2 ml Eppendorf tube and placed in a centrifuge. The SPE sorbent was conditioned with 50 μl MeOH added to the tip and the centrifugation and equilibration steps were performed at 540 *g* for 5 min at 4°C and with 50 μl of water (640 *g*, 10 min, 4°C); prepared tips were stored in a polypropylene plastic box at room temperature until use.

### Sample preparation workflow

#### Workup of the arthropod samples

The collected hemolymph was transferred directly into a 1.5 ml Eppendorf tube containing 50 μl of cold MeOH. When analyzing whole larvae or adult insects, washed individuals were placed in a 2 ml Eppendorf tube containing 100 μl of cold MeOH or more, depending on the number of individuals extracted. Then the samples were homogenized with a TissueLyser LT (Qiagen, Hilden, Germany) at −18°C, 50 Hz, for 5 min.

The following liquid-liquid extraction and oximation procedure was the same for all sample types: a 2 μl aliquot of the internal standard (IS) stock solution (100 ng ml^-1^) containing taxisterone and ajugasterone C was added to each sample to reach a final concentration of 2 ng ml^-1^. Samples were vortexed for 1 min and then sonicated in an ultrasonic bath (10°C, for 5 min). The samples were then centrifuged (4650 *g*; 5 min), and the methanolic supernatant was collected in a new vial. The extraction step with 50 μl MeOH was repeated. The combined extracts were evaporated to dryness under argon and subjected to oximation by adding 250 μl of an aqueous solution of hydroxylamine hydrochloride (100 mg mL^-1^) and heating at 70°C for 90 min (25).

The derivatized extract was then applied to the prepared stock PT/SPE tip (850 *g*, 25 min, 4°C) and subsequently washed with 50 μl of 5% MeOH (850 *g*, 10 min, 4°C). To facilitate the elution, a centrifuge is required with sufficient clearance between the lid and the vial holder to allow the PT to be inserted into the centrifuge. The receiving Eppendorf tube was exchanged for a new 1.5 ml collection tube and the ecdysteroid oximes were eluted with 50 μl 90% MeOH (850 *g*, 10 min, 4°C). Finally, the eluate was evaporated to dryness under a gentle stream of argon and redissolved in 100 μl of 30% MeOH with reserpine, the injection standard (1 ng ml-1), prior to HPLC-MS/MS analysis.

#### Human plasma, urine, and peripheral capillary blood

Plasma and urine of the test subjects were collected before and after the consumption of maral root. In addition, peripheral blood was also collected from a finger of some test subjects. The samples were processed in the Department of Clinical Chemistry of the České Budějovice Hospital, Czech Republic, under the supervision of Dr Miroslav Verner, MD. Samples were supplied in closed plastic vials and stored at 4°C until use. Aliquots of 10 μl and 50 μl of plasma and urine, respectively, were used. The volume of peripheral capillary blood collected was 10 μl. Each sample was extracted with 3 volumes of MeOH. After the extraction step, the sample preparation procedure was identical to that described above.

#### HPLC-MS/MS analysis

Quantitative analysis was performed using an HPLC 1290 LC Infinity II coupled with a triple quadrupole mass spectrometer 6495B (both Agilent Technologies, Santa Clara, CA) equipped with an ESI Jet Stream ion source and operated in positive ion MRM scan mode. A reverse phase C18 column (Zorbax eclipse plus, 50 × 3 mm, 1.7 μm, Agilent Technologies, Santa Clara, CA) was used for chromatographic separation. The mobile phase consisted of A: water (0.5 mM NH_4_F) and B: MeOH (0.5 mM NH_4_F) and the analytes were separated with the following gradient elution: 0–3 min 30–100% B, 3–6 min 100% B, and 6–7 min 30% B.

The following conditions applied for chromatography: Column temperature 40°C, flow rate 0.4 ml·min^-1^, injection volume 5 μl, autosampler temperature 10°C, and total analysis time was 7 min. All other details on the method and device settings can be found in supplemental Tables S1 and S2. The Agilent MassHunter Workstation software was used to process the acquired MRM-MS/MS data.

#### Method validation

The presented method for the quantification of 20E, E, MaA, and 2dE has been fully validated according to the US FDA guidelines (15, 16) for linearity, sensitivity, accuracy, precision, selectivity, recovery, carryover, and matrix effect. All standard solutions of ecdysteroids were prepared in MeOH at a stock concentration of 1 mg mL^−1^. The working concentrations were diluted with MeOH at concentrations of 100, 10, 1, and 0.1 ng ml^−1^ prior to preparation. Quality control (QC) samples and points of calibration curves were prepared in 50 μl MeOH with 1 μl hemolymph of the fire bug *Pyrrhocoris apterus* (to obtain a blank matrix). The hemolymph of 1-day-old adult *Pyrrhocoris apterus* was chosen as the blank matrix as there were no ecdysteroids in 1 μl volume. The samples were then treated exactly as described in the sample preparation workflow.

#### Selectivity, linearity, sensitivity, LLOQ, accuracy, and precision

Selectivity was assessed by comparing the hemolymph blank matrix (n = 6) with a pure solution spiked with IS (n = 6) and with a blank matrix spiked with ecdysteroids at the lower limit of quantification (LLOQ) and IS (n = 18). Absence of interference was accepted if the response was less than 20% of the LLOQ for the analyte and 5% for the IS. Linearity was defined within 0.01–10 ng ml^−1^ (for 20E). The LLOQs were determined according to FDA guidelines and declared as the lowest calibration points when the back-calculated concentration of the calibration was no more than 20% of the coefficient of variation (CV) (precision) and the accuracy was below 20% of the nominal concentration. The LLOQs were also used to evaluate the sensitivity of the analytical method according to the acceptance criteria. Intra-day accuracy and precision were assessed at four concentrations (LLOQ, low QC, medium QC, and high QC) with 5 replicates. Accuracy was expressed as a percentage of the nominal value and precision as the CV. The acceptance criteria for accuracy and precision were within 15%, except for LLOQ (>20%).

#### Recovery, matrix effect, autosampler stability, carryover, PT-SPE sorbent capacity, retention time, and stability

Recovery was checked by comparing the analytical results between the blank matrix spiked with analytes and IS and the pure solution spiked with analytes and IS. The recovery rate was determined for three concentrations (low, medium, and high) in five replicates. The recovery of the sample preparation procedure (including extraction, derivatization, and PT-SPE) for 20E was also performed. The synthesized 20E oxime was used for this purpose. Two preparation procedures were compared. The first was the standard preparation, derivatization of the 20E standard with IS. In the second sample, only IS was derivatized and synthetized 20E oxime was added after extraction, in the last step before evaporation and reconstitution before HPLC-MS/MS analysis. Recovery was performed in three replicates at two concentrations and expressed as a percentage.

The matrix effect was determined using the matrix factor, which is the ratio between the analyte area in the blank matrix and the area of the analyte in pure solution normalized to IS (the ratio of IS in the blank matrix to IS in pure solution). The normalized matrix factor must then not exceed the limit of 15%. We performed this evaluation at three concentrations (low, medium and high) and in five replicates.

Stability testing of ecdysteroid derivatives was performed using the blank matrix spiked with analytes at two concentrations in five replicates. Samples were freshly prepared and measured prior to analysis. The samples were then left in an autosampler (10°C) and measured again after 1, 3, 7, 24, and 72 h. This stability test was chosen to simulate our measurement conditions where samples may wait before injection into the autosampler when a large number of samples are present.

The synthesized 20E-oxime was used to evaluate the capacity of the PT sorbent. It was loaded onto the conditioned PT-SPE (250 μl of aqueous reagent), and each of the elution volumes from loading, washing, and elution were collected and measured separately. The sorbent capacity was compared at 1.25 ng and 12.5 ng levels.

Carryover was tested by injecting three consecutive blanks after the highest calibration point. The criteria were that the response for the analytes should not exceed 20% of the average response of the LLOQ or 5% of the average response of the IS. Finally, the retention time stability of the LC-MS/MS system was tested and observed over several months.

## Results

**Fig. 2:**
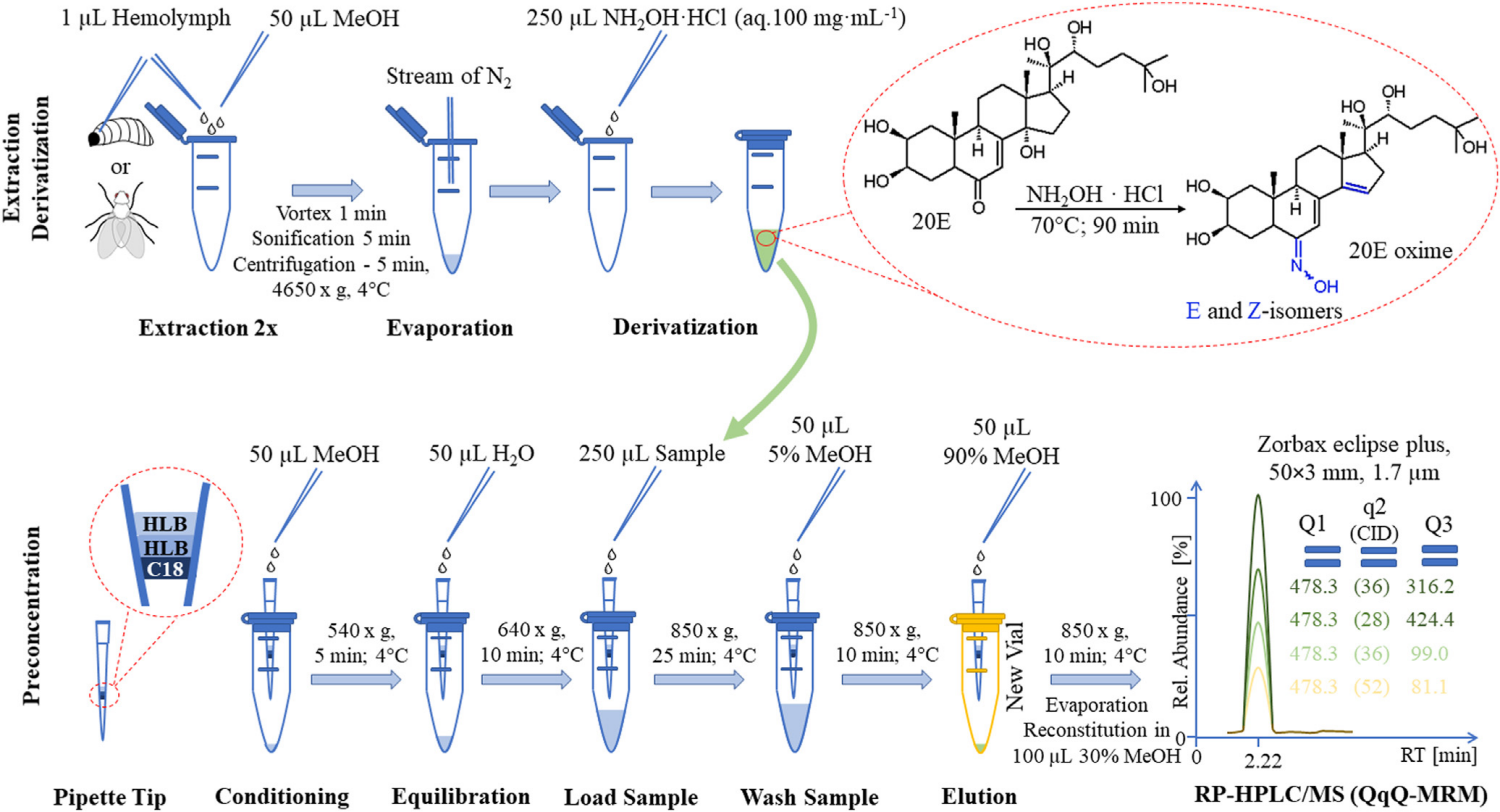
Graphical diagram of the sample preparation workflow for ecdysteroid analysis. The detailed conditions are shown for the preparation of 1 μl of insect hemolymph. The sample preparation protocol includes three main steps: (a) extraction from biological material; (b) derivatization of the main ecdysteroid 20-hydroxyecdysone (20E) with hydroxylamine; and PT-SPE purification with final MRM HPLC-MS/MS analysis on a triple quadrupole MS. The experimental conditions and MRM transitions for the 20E-oxime derivative are shown. PT-SPE, pipette tip solid-phase extraction; MRM, multiple reaction monitoring.

The graphical presentation of the developed method is depicted in Figure 2 and comprises three sample preparation steps prior to HPLC-MS/MS analysis: (A) extraction with MeOH from the biological material, (B) conversion of the ecdysteroid keto-group in ecdysteroids into oximes, (C) the PT-SPE.

### Optimization of sample preparation procedure

#### Preliminary precautions

A fully validated ultratrace analysis of ecdysteroids requires the use of highly purified laboratory materials and sophisticated measures to minimize unwanted sample matrix effects. Therefore, larvae must be washed with deionized water prior to sample preparation to remove food and body surface residues. Similarly, the hemolymph must be collected with a clean capillary and immediately placed in cold MeOH, previously prepared in a clean tube. Otherwise, the hemolymph may oxidize/melanize and turn a gray color. For larger sample quantities or many samples with high-lipid content, consider prior extraction with hexane, chloroform, or tert-butyl methyl ether to minimize undesirable matrix effects. However, if the minimal sample amount is used, this step is not necessary as it may increase the risk of less reproducible results (13, 14).

#### Derivatization

Ecdysteroids, like most other steroids, are poorly ionizable by electrospray, and to achieve sensitivity in the femtomole range, increasing the ionization efficiency by a suitable derivatization method is required. Unlike the intact aliphatic and alicyclic hydroxyl groups, the 6-oxo group is a natural target for chemical derivatization with nitrogen-containing reagents.

In our preliminary experiments, we tested Girard reagents such as Girard-P and Girard-T, as well as some hydroxylamines, especially benzylhydroxylamine or methylhydroxylamine. However, the reactivity of the 6-oxo group under the previously described conditions led to derivatives in low yield, even under harsher conditions (85°C, 4 h), and several products were observed (data not shown). An exception was the reaction with the simplest and cheapest hydroxylamine hydrochloride. At large access and in aqueous medium, it reacted readily with the 6-keto group of ecdysteroids, with concomitant dehydration at positions 14 and 15 (25, 30). We therefore synthesized the oxime of 20E and demonstrated that it increased the ESI^+^ signal by 16- to 20-fold depending on the concentration (see supplemental Table S4 in the Supplementary information for data). The high excess of hydroxylamine hydrochloride in the extraction medium overcame the low reactivity of the 6-oxo group (25). In addition, the high polarity of the oximating reagent facilitated its elution near dead volume, far from the eluted analytes in the reversed-phase separation systems used in both SPE purification and HPLC separation steps.

#### Pipette-tip solid-phase extraction

We tested different sorbent types (C8, C18, hydrophilic-lipophilic balanced sorbent, and divinylbenzene sorbent) from different SPE disk suppliers, their number, combination, and sequence of sorbent layers added to the PT, conditioning, and different elution solvents and their mixtures (data not shown). The synthetic 20E-oxime derivative was used to optimize the PT-SPE conditions. A three-layer arrangement in the PT, consisting of a C18 sorbent and two overlying hydrophilic-lipophilic balanced sorbent layers, each embedded in the cut out microdisk, proved to be an optimal compromise in terms of high analyte yields, minimized matrix effects, ease of preparation and accessibility of the sorbent. The micro disks were easily cut from commercially available disks with polytetrafluoroethylene matrix (Affinisep) and held well in the standard 200 μl tip. Evaluation of the sorption capacity showed low 20E-oxime losses at the PT-SPE tip in the low nanogram range. The application of 1.25 ng and 12.5 ng of 20E-oxime in a total volume of 250 μl of reagent resulted in a satisfactory yield of 95.2% and 83.5% of 20E, respectively.

#### HPLC-MS/MS analysis

It is well known that ecdysteroids can be readily separated on C18 LC columns (25, 27, 29). We found that the Zorbax Eclipse plus column (Agilent) was optimal for separation, with symmetrical narrow peaks and short analysis time (7 min). It is important to note that the derivatization reaction yielded E/Z oxime isomers for each analyte, which were eluted as a single peak in the optimized C18 separation system (25, 29). We tested different solvents (acetonitrile, MeOH, and water) and additives (acetic acid, ammonium formate, and ammonium fluoride); some graphical data are available in the Supplementary information in supplemental Fig. S2. Surprisingly, we found that the addition of 0.1% ammonium fluoride to the aqueous methanolic mobile phase enhanced the ESI^+^ signal of the ecdysteroid oximes, similar to that reported for some steroid analytes (testosterone, progesterone, androstenedione, or spironolactone) (32, 33, 34). The previously observed collision-induced decomposition, which resulted in neutral losses of up to four water molecules in other derivatized and natural ecdysteroids (28) was also a feature of anhydro-6-oxime derivatives. The major productions occurred upon cleavage of the C17-20 bond. The previously reported stability of vicinal C2,3-hydroxy groups at the collision-induced decomposition (28) was also observed and allowed the assignment of hydroxy group elimination to either the sterol scaffold or the side chain and was therefore a valuable option for the identification of ecdysteroids by MS/MS.

The MRM transitions of the ecdysteroid derivatives are listed in Table 1. The complete list of MRM transitions used, including the nonderivatized ecdysteroids and the injection standard (reserpine), are shown in supplemental Table S3.

**Table 1:** MRM transitions of all quantified ecdysteroids oxime derivatives, including precursor ion, product ions with collision energy, and retention time

Ecdysteroid derivative	Precursor Ion	Product Ions (CE [eV])	RT [min]
20E oxime	478.3	**316.2** (**36),** 424.4 (28), 99.0 (36)	2.227
E oxime	462.3	**444.3** (**24),** 318.2 (36), 316.2 (32)	2.492
MaA oxime	492.3	**316.1 (32),** 474.3 (24), 344.2 (32)	2.454
2dE oxime	446.3	**428.2** (**24),** 302.3 (36), 284.1 (44)	3.003
Tax oxime (IS)	462.3	**316.1** (**32),** 426.3 (32), 266.2 (52)	2.672
AjugC oxime (IS)	478.3	**332.1** (**36),** 314.2 (32), 81.0 (56)	2.670

Productions, given in bold, are used for quantification.

2dE, 2-deoxyecdysone; 20E, 20-hydroxyecdysone; AjugC, ajugasterone C; CE, collision energy; IS, internal standard; MaA, makisterone A; MRM, multiple reaction monitoring; RT, retention time; Tax, taxisterone.

#### Method validation

Ecdysteroids fluctuate in hemolymph during insect development (14), so finding a suitable ecdysteroid-free sample that is highly desirable for matrix calibration can be challenging. After extensive testing of various hemolymphs collected from the major insect models studied in-house at the Biology Centre, we found that hemolymph from the 1-day-old adult insect *Pyrrhocoris apterus* contains no detectable levels of ecdysteroids. This selected matrix is also biologically close to studied samples, unlike other matrices used in various studies, for example, extraction buffer solution (24) and fetal bovine serum (11), to compensate for the matrix effect. In addition, the larger body of *Pyrrhocoris apterus* (in contrast to Drosophila) contained larger amounts of hemolymph and allowed for aliquoting, which is essential for proper method validation. The choice of an appropriate IS was another important point in the method validation process. Since stable isotope-labeled ecdysteroids were unavailable and their commercial synthesis would be very expensive, we tested two ISs, taxisterone and ajugasterone C. Taxisterone was chosen for quantification, while ajugasterone C served as a complementary IS useful for estimating unexpected matrix effects. These compounds are phytoecdysteroids that have been detected mainly in plants (e.g., most frequently in *Leuzea carthamoides*) (7, 30) or to a lesser extent in insect (*Bombyx mori*) ovaries (taxisterone) (35) and in marine corals (ajugasterone C) (36).

#### Selectivity, linearity, LLOQ, accuracy, and precision

No interference of ISs (taxisterone, ajugasterone C) was observed in the MRM MS/MS chromatograms obtained by analysis of the blank hemolymph matrix. The quantified ecdysteroids also exhibited minimal interfering peaks, such that the response did not exceed 20% of LLOQ.

The responses for all ecdysteroid analytes were linear within a given range, and the coefficient of determination *R*^2^ obtained from linear regression was higher than 0.997 for all analytes. The calibration plot was weighted using the 1/x method. The calibration line was linear above 100 ng ml^-1^, but the risk of HPLC-MS/MS instrument contamination at higher concentrations was observed. The capacity of the three-layer sorbent in the PT-SPE was in a maximum calibration range of about 5–10 ng·ml^-1^ but the linearity of the calibration curve above these values could be additionally compensated for by the use of the ISs. For 20E, the calibration range was 10–5000 pg ng·ml^-1^.

The lower limits of quantification for each ecdysteroid together with precision and accuracy are summarized in Table 2.

**Table 2:** Validation parameters (for n = 5) measured and calculated for method validation according to FDA guidelines

Analyte	Nominal concentration [ng·mL^-1^ (fmol·ml^-1^)]		Accuracy [%]	Precision [%]	Concentration level [ng·ml^-1^ (pmol·ml^-1^)]		Recovery [%]	ME [%]
20E	LLOQ	0.01/0.02	89.2	4.5	Add.1	0.25 (0.52)	103.4	3.2
	Low QC	0.03 (0.06)	102.7	7.9	Add.2	1.0 (2.0)	114.1	13.9
	Med. QC	0.25 (0.52)	100.0	8.8	Add.3	10.0 (20.8)	108.8	8.3
	High QC	5.00 (10.4)	111.0	2.7				
E	LLOQ	0.1 (0.2)	116.0	8.8	Add.1	0.25 (0.52)	100.6	0.5
	Low QC	0.3 (0.6)	114.6	2.4	Add.2	1.0 (2.1)	112.2	12.2
	Med. QC	1.0 (2.1)	99.9	10.3	Add.3	10.0 (20.8)	96.3	4.3
	High QC	5.0 (10.4)	92.7	11.5				
MaA	LLOQ	0.05 (0.10)	101.6	8.3	Add.1	0.25 (0.52)	113.3	12.5
	Low QC	0.15 (0.31)	113.2	5.9	Add.2	1.0 (2.1)	114.9	13.0
	Med. QC	1.0 (2.1)	108.7	4.5	Add.3	10.0 (20.8)	108.5	7.9
	High QC	5.0 (10.4)	103.1	7.7				
2dE	LLOQ	0.025 (0.05)	89.0	8.4	Add.1	0.25 (0.52)	116.1	22.2
	Low QC	0.075 (0.16)	98.7	2.8	Add.2	1.0 (2.1)	117.8	25.2
	Med. QC	0.5 (1.04)	107.5	8.1	Add.3	10.0 (20.8)	119.9	19.8
	High QC	5.0 (10.4)	101.5	5.2				

Concentrations are expressed in ng·ml^-1^ and in pmol·ml^-1^. The table contains information on accuracy, precision, recovery, and matrix effect. The values are defined for the resulting ecdysteroid derivatives (oximes).

2dE, 2-deoxyecdysone; 20E, 20-hydroxyecdysone; E, ecdysone; FDA, Food and Drug Administration; LLOQ, lower limit of quantification; MaA, makisterone A; ME, matrix effect; QC, Quality control.

The three-step method was somewhat laborious in terms of sample preparation, as some precautions were required. However, the analysis time was very short (7 min) and in our hands, one person could easily prepare and measure 48 samples per day.

#### Recovery

The recovery values for each ecdysteroid at three concentration levels are shown in Table 2. In most cases, the recoveries were above 100%, with 2dE having higher recoveries than the other ecdysteroids, but the CV did not exceed 15%.

Recovery of the sample was estimated from the synthesized 20E-oxime. It was in the range of 84–88%, measured at two concentrations. This result represents a very good recovery of the sample when all steps such as derivatization and PT-SPE are considered.

#### Matrix effect, stability, carry over, and retention time stability

We further evaluated the matrix effect, and the evaluated matrix effect met the established criteria (below 15%) for all analytes except 2dE-oxime, which ranged from 19.8% to 22.2% (Table 2). This may indicate that the taxisterone IS used was not particularly suitable for the quantification of 2dE-oxime due to the absence of the OH group at position 2. We also observed a lower stability of this oxime than other ecdysteroid derivatives. This problem could be circumvented by synthesizing a 20E analog with 2–4 stable isotopes in its structure.

The autosampler stability of ecdysteroid oximes was tested at two concentrations, five replicates, and over a period of 1–72 h. This is the time the samples can spend in an autosampler or refrigerator until the next day before analysis. Under routine conditions, the samples are measured immediately after preparation, and due to the short analysis time, the samples do not remain in the autosampler for long. Stability is reported in the supplementary information (supplemental Fig. S3) and shows a very low degradation of 20E-oxime and a higher degradation of 2dE-oxime, especially at a low concentration.

During the carry over test, no analyte signals were observed in the blank injected after the highest concentration.

The retention time stability was observed over a period of more than one month and it never exceeded the relative standard deviation of 0.64%.

#### Application of the developed method

**Fig. 3:**
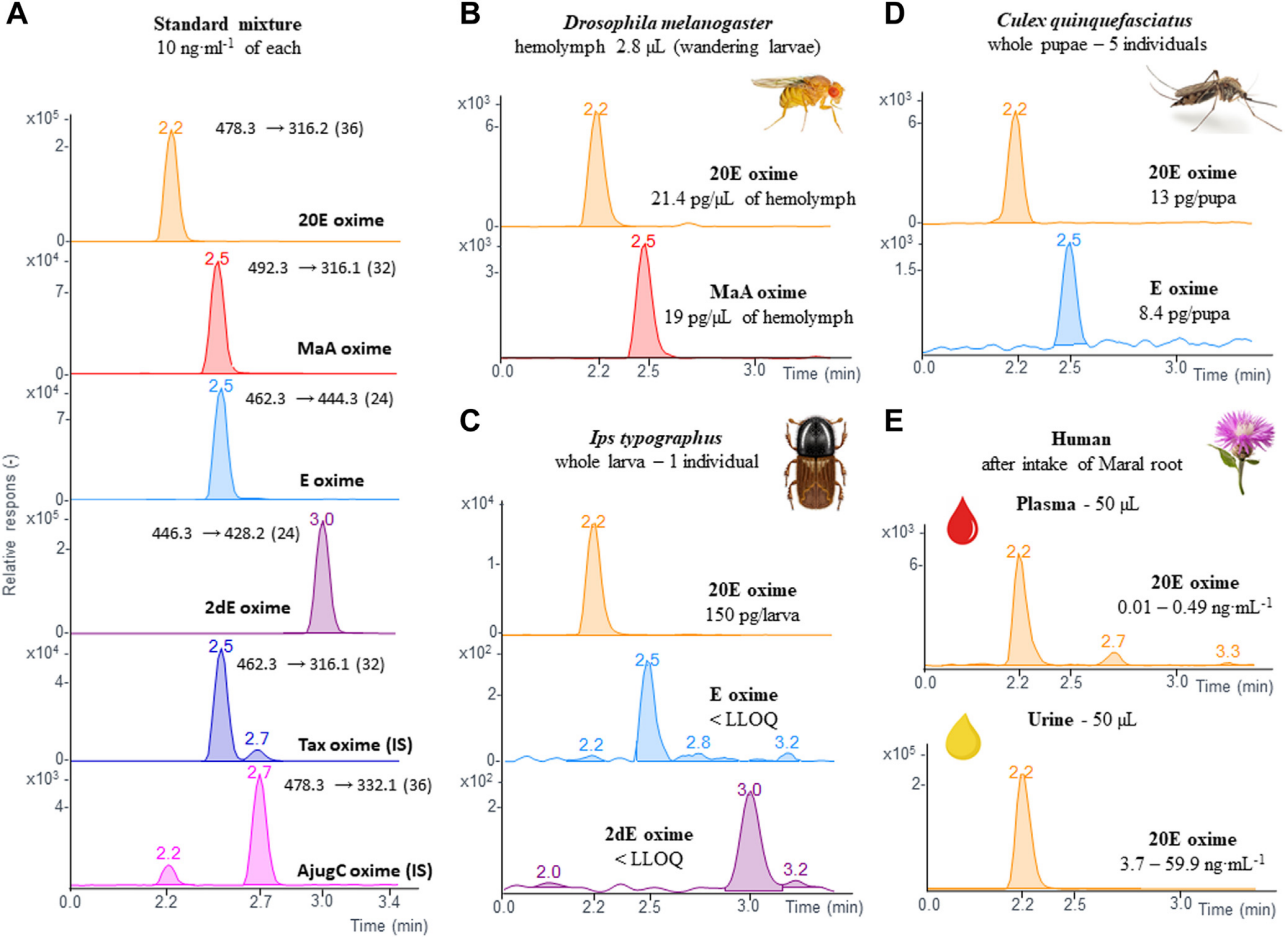
MRM LC-MS/MS chromatograms for all ecdysteroid oximes studied. The chromatograms shown were used for quantification, and the relevant transitions, including the collision energy [eV], are also indicated. A: Standard mixture of ecdysteroids with two internal standards, ajugasterone C (AjugC), and taxisterone (Tax). B: 20-hydroxyecdysone (20E) and makisterone A (MaA), analytes from the hemolymph of *Drosophila melanogaster*. C: 20E, ecdysone (E), and 2-deoxyecdyosone (2dE) found in the larva of *Ips typographus* (bark beetle) at the concentrations described. D: 20E, E, and 2dE in the mosquito *Culex quinquefasciatus* at the concentrations indicated. E: Chromatograms obtained for 20E and concentrations measured in human urine and plasma after oral ingestion of maral root (ecdysteroid-rich plant). MRM, multiple reaction monitoring; 2dE, 2-deoxyecdysone; E, ecdysone.

The new validated method was investigated for the quantification of ecdysteroids in minimal amounts of biological material, especially in important arthropod models, and also in human biofluids. The representative MRM HPLC-MS/MS chromatograms in Figure 3 show the detection of ecdysteroid profiles: A) in a standard mixture, B) in the hemolymph of a single larva of *Drosophila* (a versatile model organism), C) in the whole larva of the important forest pest bark beetle *Ips typographus*, D) in five collected pupae of the mosquito *Culex quinquefasciatus*, and E) in human plasma and urine after consumption of maral root, a rich source of 20E.

#### Arthropod samples

First, we investigated the newly developed analytical approach to study ecdysteroids in the model organism *Drosophila melanogaster*. Adult animals, larvae at different stages and pupae, whole individuals or hemolymph, and even body parts of Drosophila were measured, for example, collected brains or the rest of the body after hemolymph collection. In addition, different hemolymph extractions were tested, such as standard hemolymph collection, perfusion, or homogenization of whole individuals. The results are shown in Table 3.

**Table 3:** Ecdysteroid contents in different insect species and types of samples measured during the study

Animal	Development stage	Sample type	Sample amount	20E concentrations	Other EEcdysteroids concentrations
Drosophila melanogaster	Prepupa	Hemolymph	∼ 0.3 μl (from 1 individual)	27.8 pg/μL	MaA: 7.1 pg/μl
		Whole larva	1 individual (∼2 mg)	27.4 pg/larva	MaA: 17.3 pg/larva
		Larva - body without hem.	1 individual	38.7 pg/larva	MaA: 24.5 pg/larva (rest of body)
		Brain	10 brains	4.3 pg/brain	NF
	Wandering larvae	Hemolymph	2.8 μl (from 10 larvae)	21.4 pg/μl	MaA: 19 pg/μl hemolymph
	Adult (3 days old)	Perfused hem.	3, 5, and 10 perfused individuals	0.5 pg/adult	NF
		Whole adults	3 and 10 individuals	0.3 pg/adult	NF
Ips typographus	Larvae	Hemolymph	1 μl	11 pg/μl hem.	E, 2dE < LLOQ
		Whole larva	1 individual	∼ 150 pg/larva	E, 2dE < LLOQ
Pityogenes chalcographus	Larvae	Whole larva	1 individual	44.8 pg/larva	NF
Culex quinquefasciatus	Last instar	Whole larva	1 individual	11 pg/larva	NF
		Whole pupae	1, 3, 5	13 pg/pupa	E: 8.4 pg/pupa
		Adult females	>10 individuals	0.2 pg/adult	E < LLOQ
		Adult males	>10 individuals	<LLOQ	NF
Ixodes ricinus	Adult	Hemolymph	2.5 μl	9.6 8 pg/μl	NF
		Ovaries	5 ovaries	27.7 pg/5ov	MaA < LLOQ

Information is provided on the developmental stage of each insect species, the part of the insect used, the volume or amount of the sample and the resulting concentration of 20E or other ecdysteroids.

2dE, 2-deoxyecdysone; 20E, 20-hydroxyecdysone; E, ecdysone; hem, hemolymph; LLOQ, lower limit of quantification; MaA, makisterone; NF, not found; ov, ovaries.

The suitability of the method was further demonstrated by the analysis of ecdysteroids in a whole larva and in the hemolymph of a single larva of two bark beetle species, *Ips typographus* and *Pytiogenes chalcographus*, two difficult spruce pests in Europe. Nothing is known about the occurrence of ecdysteroids in bark beetles. Nevertheless, 20E was detected and quantified in both organisms, while in *Ips typographus* E and 2dE were found below the LLOQ.

Homogenized whole larvae or pupae of the mosquito *Culex quinquefasciatus* in the last instar contained about 10 pg/animal. Interestingly, ecdysteroids were not detected in the homogenized adult mosquitoes (n < 5). When a higher number of individuals (n > 10) were processed for analysis, 20E and E (below the LLOQ) were only detected in the females. In this case, the higher amount of biological material used showed pronounced matrix interference. The additional methyl-terc.butylmethylether defatting step was performed after homogenization of the animals to minimize unwanted matrix effects.

Another positive ecdysteroid analysis was performed in the tick *Ixodes ricinus*, where 20E was found in the hemolymph and ovaries, with Ma Also detected below the LLOQ. The ecdysteroid concentrations found in all these samples are listed in Table 3.

#### Human plasma and urine

We monitored major 20E in human plasma, urine and peripheral capillary blood in seven objects that consumed grounded maral root for 14 days prior to body fluid collection. Interestingly, all the respondents had no measurable 20E in serum or urine before the regular maral root consumption. The results are summarized in Table 4 and indicate that ecdysteroids did not circulate at measurable levels in subjects not consuming food rich in 20E and that the 20E was excreted in urine, where the 20E levels quickly became higher than those in plasma.

**Table 4:** Concentration values of 20E in human plasma, urine, and capillary blood from a finger after 14-day oral ingestion of a dietary supplement from maral root

Subject	Plasma [ng·ml^-1^ (nmol·l^-1^)]	Urine [ng·ml^-1^ (nmol·l^-1^)]	Peripheral capillary blood [ng·ml^-1^ (nmol·l^-1^)]
Male 1	0.20 (0.43)	59.9 (124.9)	0.81 (1.69)
Male 2	0.32 (0.66)	55.8 (116.2)	<LLOQ
Male 3	0.01 (0.02)	14.5 (30.2)	0.02 (0.04)
Female 1	0.08 (0.16)	11.8 (24.5)	<LLOQ
Female 2	0.49 (1.03)	38.3 (79.8)	0.76 (1.59)
Female 3	0.3 (0.06)	3.7 (7.7)	0.46 (0.96)
Female 4	0.3 (0.06)	10.6 (22.0)	0.02 (0.05)

No detectable 20E values were measured in the same subjects before supplementation of the maral root.

20E, 20-hydroxyecdysone; LLOQ, lower limit of quantification.

## Discussion

The analytical work presented here reports on an improved and validated method for the analysis of ecdysteroids in very small samples. The developed protocol allowed the quantification of 20E at low picogram concentrations in a submicroliter volume of *Drosophila* hemolymph, whole larvae, adult insects, and even in isolated brains (Table 3). In addition, the method proved to be suitable for the detection and quantification of ecdysteroids in hemolymph and body of other small important organisms studied, such as bark beetle *Ips typographus*, *Pityogenes chalcographus*, mosquito *Culex quinquefasciatus*, and tick *Ixodes ricinus* (Table 3).

Table 5 gives an overview of the analytical methods reported for the quantification of ecdysteroids in animals. To our knowledge, the new approach described is the most sensitive validated method reported. A fully validated, robust method for the quantification of ecdysteroids in biological material derived from small organisms such as many arthropods has been lacking, and the hormone analysis has been validated in the past mainly for human or equine body fluids (6, 21, 24, 25, 27, 34).

**Table 5:** Overview of the analytical methods reported for the quantification of ecdysteroids in animals

Source	Analytes	(L)LOQ or LOD	Sample amount	Analytical methods	Ref.
Arthropods					
*Drosophila melanogaster*	20E	5 pg·ml^-1^ (LOD)	6–200 crustacean individuals (depending on instar)	HPLC-MS/MS, (QqQ-MRM, t-SIM)	(11)
	E, MaA, 24methylE, 24epi-methylE, 24epiMaA			Methanol extraction- > LLE (MeOH/hexane)	
*Drosophila melanogaster*	20E	10 fmol per inj.(LOD)	10 larvae	NanoLC-MS/MS (Qtrap, LTQ-Orbitrap), EIA	(13)
	E, MaA	- E equivalents		MeOH extr- > LLE MeOH/CHCl_3_	
	24epiMaA				
	20dMaA				
*Drosophila melanogaster*	20E	16 pg/tube (LOQ)	10 whole larvae	EIA	(37)
	E	1.8 pg/tube (LOQ)	6 ring glands	MeOH extraction	
*Aedes aegypti*	20E	84.2 pg (LOD)	1, 5, 10; > 50 larvae	EIA - quantification	(38)
	E	4.5 pg (LOD)	1, 5, and 10 adults females	HPLC-UV-MS -qualification	
	PonA	NA		MeOH extr., SPE C18 or LLE (BuOH, water/CHCl_3_)	
	MaA	NA			
*Bombyx mori*	20E	0.1 ng ml^-1^ (LLOQ)	50 μl hem., 2x prothoracic glands	HPLC-MS/MS (QqQ-MRM)	(24)
	E	0.1 ng ml^-1^ (LLOQ)		LLE (aq./BuOH)	
	2dE	0.25 ng ml^-1^ (LLOQ)			
*Spodoptera littoralis*, *Schistocerca gregaria*, *Cancer pagurus*	20E	500 pg per inj. (LOD)	Hem.	HPLC-APCI-MS (Q)	(39)
	E	10 pg per inj. (LOD)	Eggs and hem.	HPLC-RIA	
	MaA (ISTD)	(5 μl injection)	Ovary tissue (amount not specified)	Ethanol, methanol extr. - > SPE C18	
*Daphnia Magna*	20E	230 pg·ml^-1^ (LLOQ)	25–100 adults juveniles and neonates)	UPLC-MS/MS (SRM, QqQ) Deriv. (hydroxylamine).	(25)
	E	210 pg·ml^-1^ (LLOQ)		>LLE (aq./MTBE)	
	PonA	380 pg·ml^-1^ (LLOQ)			
Ecdysteroids standards	E	50–100 pg per inj. (LOD)	-	GC/MS (Q)	(21)
	AjugC, PolyB			LC-MS/MS (QqQ)	
Vertebrates					
Equine urine and plasma	20E	200 pg ml^-1^ (LOD)	1 ml of equine plasma or urine	HPC-MS/MS, HPLC-HRMS	(6)
	PonA				
	Tur				
	E				
	AjugC				
Human serum	20E	0.06 ng ml^-1^ (LOD)	480 μl of serum	UHPLC-MS/MS (QqQ-MRM)	(27)
	E	0.14 ng ml^-1^ (LOQ)		SPE (HLB) - 60 mg per cartridge	
	PonA (ISTD)	NA			
		-			
Mice, rats: stomach, small and large intestines, feces, bile, urine, plasma.	20E	2.5 ng ml^-1^ (LOQ)	5g of faeces	HPLC-MS/MS	(26)
	Post 14dPost	10 ng ml^-1^ (LOQ)	Other amounts not specified	Ethanol extraction	
	14d20E	100 ng ml^-1^ (LOQ)		C18 SPE	
	20RSPost	1 ng ml^-1^ (LOQ)			
	6OH20E	100 ng ml^-1^ (LOQ)			
	6OH14d20E	5 ng ml^-1^ (LOQ)			
	PolyB (ISTD)	2.5 ng ml^-1^ (LOQ)			
Calf urine and feces	20E	0.5 μg l^-1^	5 ml of urine	HPLC-MS/MS (QqQ)	(23)
			2 g of feces	SPE C18 - 2 g per cartrige and 1 g of silica	

The measured ecdysteroids and their (L)LOQs and LODs, including the sample amount and analytical method approach are listed where available.

6OH14d20E, 6-hydroxy-14-deoxy-20-hydroxyecdysone; 6OH20E, 6-hydroxy-20-hydroxyecdysone; 14dPost, 14-deoxypoststerone; 14d20E, 14-deoxy-20-hydroxyecdysone; 20RSPost, 20-(*RS*)-dihydropoststerone; 24epiMaA, 24-epi-makisterone A; 24epi-methylE, 24-epi-methyl-ecdysone; 24methylE, 24-methyl-ecdysone; AjugC, ajugasterone C; APCI, atmospheric pressure ionization; E, ecdysone; HLB, hydrophilic-lipophilic balanced sorbent; ISTD, internal standard; (L)LOQ, lower limit of quantification; LOD, lower limit of detection; MRM, multiple reaction monitoring; MTBE, methyl-terc.butylmethylether; PonA, ponasterone A; PolyB, polypodine B; Post, poststerone; SPE, solid-phase extraction; Tur, turkesterone.

The described ultratrace MRM-LC-MS/MS method was fully validated and proved to be highly specific, robust, and sensitive for the quantification of the four major free ecdysteroids (20E, ecdysone, MaA, and 2-deoxyecdysone) in less than 1 μl hemolymph of the arthropods studied. The desired LLOQs for the four hormones (20E, E, MaE, and 2dE) were 0.01; 0.1; 0.05; 0.025 pg·mL^-1^ (20; 200; 100; 50 fmol mL^-1^), respectively, with very good accuracy, precision (relative standard deviation <15%) and recoveries (96%–119.9%). The robust protocol presented here achieves LLOQs that are at least an order of magnitude better and enables quantification of the four main animal ecdysteroids in a single small individual (see reference data in Tables 3 and 5).

Ecdysteroids, especially 20E, are consumed as dietary supplements due to their anabolic and other positive medical effects. They are currently used by athletes and also horses as legal doping agents, and it is beneficial to have a method to regularly monitor their ultratrace levels in their body fluids (5, 6). The performance of the method was also therefore investigated by analyzing 20E in human plasma, peripheral capillary blood, and urine samples. It was shown that 20E was not present in measurable concentrations in these samples, provided that the subjects did not consume ecdysteroid-containing foods or supplements, and that 10–50 μl of biofluid may be sufficient for regular doping controls.

Finally, the new method meets the demanding requirements of researchers who want to study hormonal cycles in a subminimal amount of biological material available from lower organisms and the associated molecular mechanisms in conjunction with molecular biology approaches and omics tools.

## Data availability

This article contains supplementary data. The data supporting the results of this study are also available on reasonable request from the corresponding author (Dr Petr Šimek, simek@bclab.eu).

## Supplemental data

This article contains supplemental data.
