# Supplementary material for: A validated HPLC-MS/MS method for the simultaneous determination of ecdysteroid hormones in subminimal amounts of biological material
Source: J Lipid Res. 2024 Sep 5;65(10):100640. doi: 10.1016/j.jlr.2024.100640 (PMC11913796; doi:10.1016/j.jlr.2024.100640)
Supplement: Supplementary Tables S1–S4 and Figures S1–S3 [file mmc1.docx]

**Supplementary information**

**Table S1**

Chromatographic parameters of the HPLC system Agilent 1290 LC Infinity II for the analysis of ecdysteroids

| Parameter |  | HPLC |
| --- | --- | --- |
| Chromatographic column |  | Zorbax Eclipse Plus C18, 50×3 mm, 1,8 µm |
| Autosampler temperature |  | 10 °C |
| Column temperature |  | 40 °C |
| Flow rate |  | 400 µL/min |
| Mobile phases |  | A: H_2_O: 0.5mM NH_4_F |
| Gradient |  | B: methanol: 0.5mM NH_4_F |
|  |  | 0-3 min: 30-100% B |
|  |  | 3-6 min: 100% B  6.01-7 min: 30% B |
| Injection volume |  | 5 µL |
| Offset |  | 1.2 mm |
| Needle wash |  | Multi-wash |

**Table S2**

MS/MS parameters for the triple quadrupole (QqO) mass spectrometer Agilent 6495B operating in the MRM mode

| Parameter | QqQ |
| --- | --- |
| Mode | MRM |
| Ionisation | ESI+ |
| Gas temperature | 230 °C |
| Gas flow | 19L/min |
| Nebulizer | 40 psi |
| Sheath gas flow | 12L/min |

**Table S3**

MRM transitions of all analytes examined in this study, including retention times (RT), precursor and product ions, and corresponding collision energies (product ions in bold are transitions used for quantification). Some of the non-derivatized analytes were difficult to detect, so some unusual precursor ions are also listed (2dE and E).

| Analyte | RT [min] | Precursor Ion [M+H]^+^ | Product ions | CE [V] |
| --- | --- | --- | --- | --- |
| 20E | 2.044 | 481.3 | **445.2** | 12 |
|  |  |  | 371.3 | 12 |
|  |  |  | 165.0 | 28 |
|  |  |  | 81.0 | 56 |
| 20E oxime | 2.227 | 478.3 | **316.2** | 36 |
|  |  |  | 424.4 | 28 |
|  |  |  | 99.0 | 36 |
|  |  |  | 81.1 | 52 |
| E | 2.287 | 465.3  447.31 [M-H_2_O+H]^+^ | **429.3**  447.2  **109.1** | 12  8  28 |
|  |  |  | 429.3 | 16 |
| E oxime | 2.492 | 462.3 | **444.3** | 24 |
|  |  |  | 318.2 | 36 |
|  |  |  | 316.2 | 32 |
|  |  |  | 300.2 | 44 |
| MaA | 2.221 | 495.3 | **459.3** | 12 |
|  |  |  | 371.1 | 12 |
|  |  |  | 95.0 | 48 |
| MaA oxime | 2.454 | 492.3 | **316.1** | 32 |
|  |  |  | 474.3 | 24 |
|  |  |  | 344.2 | 32 |
|  |  |  | 94.9 | 44 |
| 2dE | 2.687 | 431.3 [M-H_2_O+H]^+^  413.3 [M-2H_2_O+H]^+^ | **413.0**  109.3 | 12  32 |
|  |  |  | **109.3**  99.3 | 18  16 |
| 2dE oxime | 3.003 | 446.3 | **428.2** | 24 |
|  |  |  | 302.3 | 36 |
|  |  |  | 284.1 | 44 |
| Tax | 2.467 | 465.3 | 429.3 | 16 |
|  |  |  | 109.0 | 20 |
| Tax oxime | 2.672 | 462.3 | **316.1** | 32 |
|  |  |  | 426.3 | 32 |
|  |  |  | 266.2 | 52 |
| AjugC | 2.367 | 481.3 | **445** | **12** |
|  |  |  | 299.2 | 24 |
|  |  |  | 189.0 | 28 |
|  |  |  | 81.1 | 52 |
| AjugC oxime | 2.670 | 478.3 | **332.1** | 36 |
|  |  |  | 314.2 | 32 |
|  |  |  | 81.1 | 56 |
| Reserpine | 3.334 | 609.3 | **195** | 28 |
|  |  |  | 236.2 | 44 |
|  |  |  | 397.2 | 44 |
|  |  |  | 174.1 | 56 |

**Table S4**

+ESI signal enhancement after the derivatization of 20-hydroxyecdysone with hydroxylamine

| Abrev. | Common name | Concentration  [ng·mL^-1^] | Response [Area] | Signal enhancement |
| --- | --- | --- | --- | --- |
| 20E | 20-hydroxyecdysone | 0.5 | 1 608 |  |
|  |  | 10 | 36 127 |  |
| 20E oxime | 14,15-anhydro-20-hydroxyecdysone oxime | 0.5 | 33 077 | 20.6x |
|  |  | 10 | 587 823 | 16.2x |


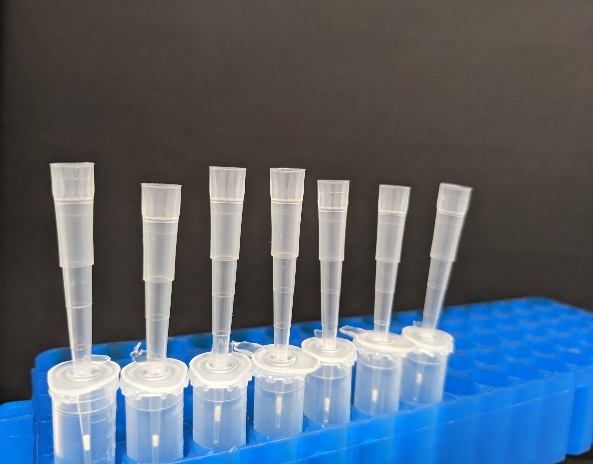

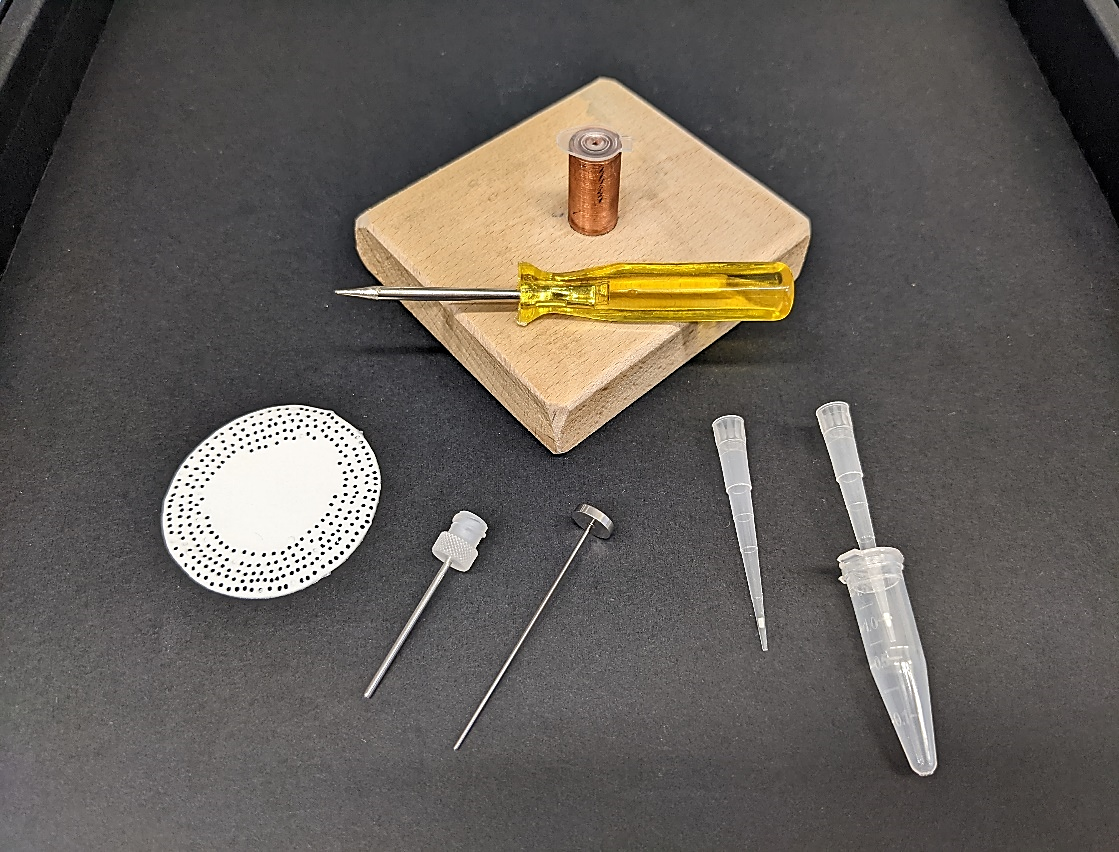

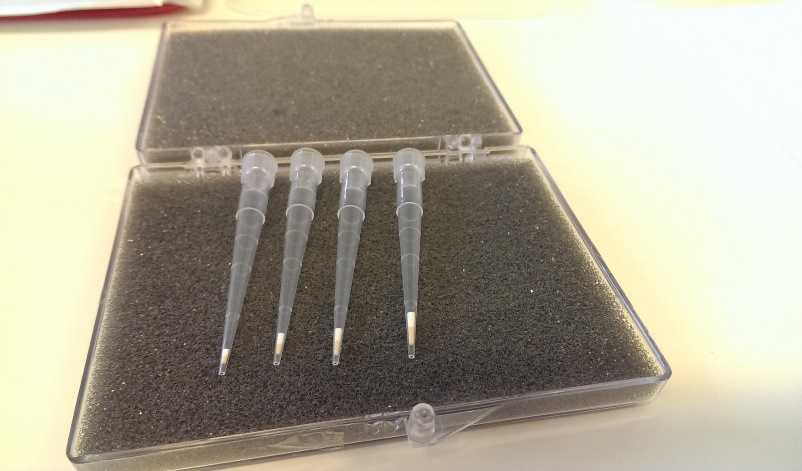


**1.**

**2.**

a)

**3.**

b)

e)

c)

d)

f)


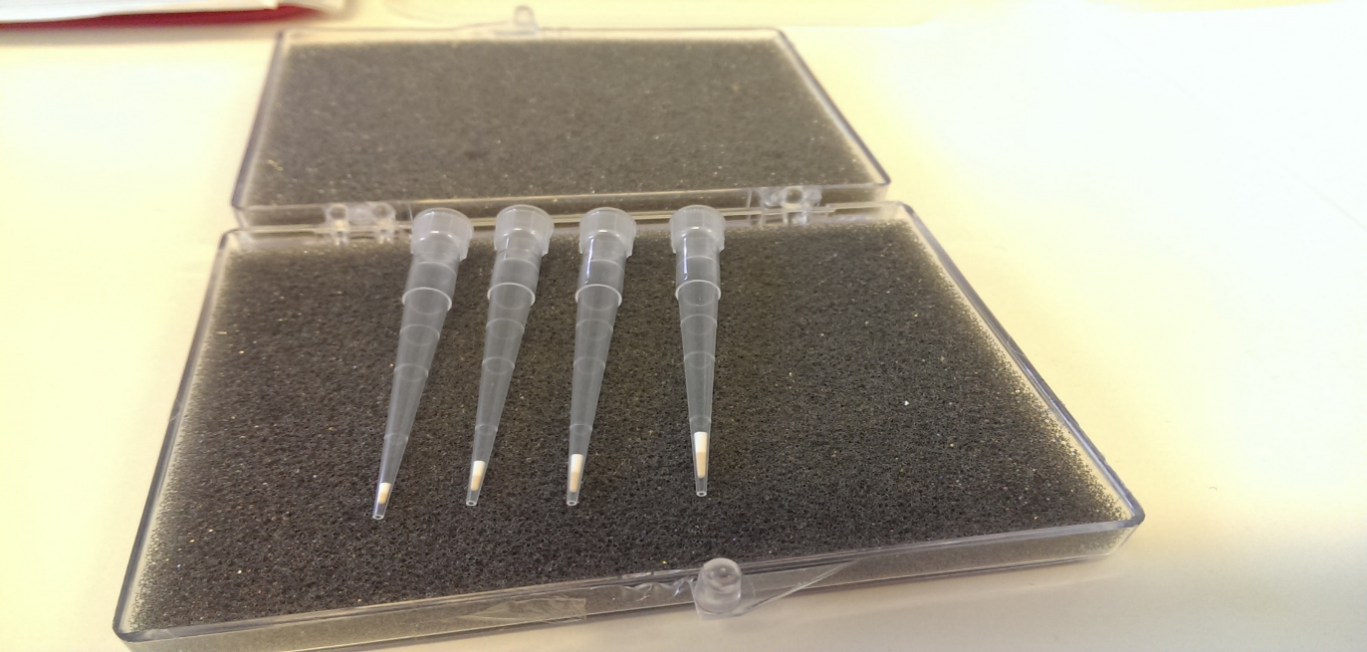


**Fig. S1.**

Tools for the preparation of PT-SPE tips: 1. a) SPE disk b) Cutter c) Plunger for removing the cut-out sorbent d) Pipette tip with cut-out piece of SPE sorbent inside e) Fully prepared SPE pipette tip in the Eppendorf tube f) Tools for piercing the lids of Eppendorf tubes. 2. Example of a multilayer PT-SPE with details. 3. SPE pipette tips in Eppendorf tubes.

**
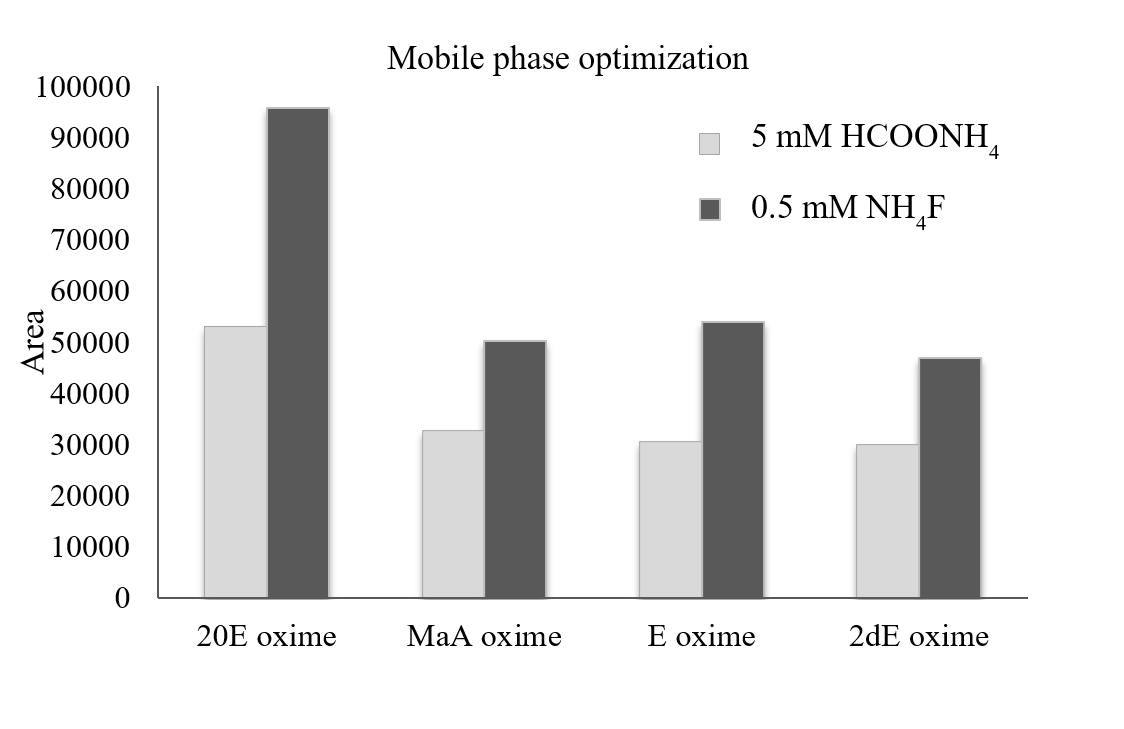
**

**Fig. S2.**

HPLC-MS optimization of the mobile phase composition – diagram of signal enhancement using 5mM HCOONH4 or 0.5mM NH4F. Ammonium fluoride shows a significant increase in signal for all analytes.

.

a)

b)

**Figure S3**

Stability diagram of four ecdysteroid derivatives, observed over 72 hours in the autosampler: a) at the concentration 10 ng·mL-1; b) at the concentration 0.5 ng·mL-1. The data are given in peak areas, the samples were measured in one batch and interspersed with QC samples at equal intervals.
